# Benefits of Short-Term (4-Week) Daily Walnut Consumption in Middle-Aged Adults at Risk for Metabolic Syndrome: Outcomes of a Randomized Controlled Trial

**DOI:** 10.3390/nu17132072

**Published:** 2025-06-21

**Authors:** Letiția Mateș, Doina Albert-Ani, Ionel Fizeșan, Andreea-Elena Petru, Roxana Banc, Marius Emil Rusu, Carmen Costache, Lorena Filip, Daniela-Saveta Popa, Daniel-Corneliu Leucuța

**Affiliations:** 1 Department of Toxicology, Faculty of Pharmacy, Iuliu Hatieganu University of Medicine and Pharmacy, 8 Victor Babes, 400012 Cluj-Napoca, Romania; micu.letitia@umfcluj.ro (L.M.); ionel.fizesan@umfcluj.ro (I.F.); petruandreeaelena@gmail.com (A.-E.P.); dpopa@umfcluj.ro (D.-S.P.); 2 Clinic of Occupational Medicine, Cluj County Emergency Clinical Hospital, 3-5 Clinicilor Street, 400006 Cluj-Napoca, Romania; doinapirvalbert@gmail.com; 3 Department of Bromatology, Hygiene, Nutrition, Faculty of Pharmacy, Iuliu Hatieganu University of Medicine and Pharmacy, 6 Pasteur Street, 400349 Cluj-Napoca, Romania; lfilip@umfcluj.ro; 4 Department of Pharmaceutical Technology and Biopharmaceutics, Faculty of Pharmacy, Iuliu Hatieganu University of Medicine and Pharmacy, 8 Victor Babes, 400012 Cluj-Napoca, Romania; rusu.marius@umfcluj.ro; 5 Department of Microbiology, Faculty of Medicine, Iuliu Hatieganu University of Medicine and Pharmacy, 400012 Cluj-Napoca, Romania; anca.costache@umfcluj.ro; 6 County Emergency Hospital Cluj-Napoca, 3-5 Clinicilor Street, 400006 Cluj-Napoca, Romania; 7 Academy of Romanian Scientists (AOSR), 3 Ilfov Street, 050044 Bucharest, Romania; 8 Department of Medical Informatics and Biostatistics, Faculty of Medicine, Iuliu Hatieganu University of Medicine and Pharmacy, 6 Pasteur Street, 400012 Cluj-Napoca, Romania; dleucuta@umfcluj.ro

**Keywords:** *Juglans regia*, endothelial function, chronic inflammation, lipid profile, cholesterol, triglycerides, blood pressure, glycemic profile, fasting blood glucose, hypertension, dyslipidemia, glycemia, waist circumference

## Abstract

**Background:** Epidemiological research has shown that regular walnut (from *Juglans regia* L.) consumption is associated with a reduced risk of cardiovascular disease (CVD), potentially attributable to their antioxidant and anti-inflammatory properties. The vascular cellular adhesion molecule-1 (VCAM-1), a protein upregulated in CVD, has been previously examined in relation to walnut consumption. However, the clinical findings regarding the effects of walnuts on endothelial function among middle-aged individuals susceptible to metabolic syndrome (MetS) remain inconclusive. **Objective:** This study examined the effects of daily walnut consumption over a four-week period on cardiometabolic parameters (lipid and glycemic profiles, as well as soluble VCAM-1 levels) and anthropometric measurements in middle-aged individuals with at least one altered MetS parameter and no medication. **Methods:** In a randomized controlled cross-over trial, 22 eligible Caucasian participants (48.81 ± 4.3 years) were selected and randomly assigned to receive either 45 g of walnuts per day or no walnuts within a controlled diet. There were two 28-day intervention periods, with a one-month washout period in between. Clinical and biochemical evaluations were conducted at the beginning and end of each intervention period. **Results:** A total of 20 participants completed the intervention and were analyzed, with walnuts being well tolerated. A significant decrease in waist circumference (*p* = 0.049) and a slight change in fasting blood glucose (*p* = 0.089) were noted following walnut intake. **Conclusions:** Short-term (4 weeks) dietary supplementation with walnuts resulted in a statistically significant reduction in waist circumference while not impacting the overall health status of participants. Longer-term studies are necessary to investigate the benefits of daily walnut consumption and its impact on the onset and development of MetS in this age group.

## 1. Introduction

Metabolic syndrome (MetS) comprises a group of comorbidities, including abdominal obesity, hypertension, dyslipidemia, and hyperglycemia [1]. Cardiometabolic risk (CMR) factors collectively refer to these conditions that directly increase the likelihood of developing metabolic disorders, such as type 2 diabetes (T2D) and cardiovascular disease (CVD). Additionally, tobacco smoking, excessive alcohol consumption, physical inactivity, unhealthy nutrition, and air pollution are considerable contributors to CMR [2].

The global incidence of MetS affects around 25% of the worldwide population, and this tendency closely aligns with the prevalence of obesity, the development of T2D, and the aging process [3]. However, the early diagnosis and treatment of dyslipidemia and hypertension have been shown to delay the onset of MetS, even with the rising prevalence of obesity [4]. The pathophysiology of MetS is intricate, involving a complex relationship between various genetic and environmental factors. Notably, chronic low-grade inflammation and neurohormonal activation play significant roles in its development. MetS is associated with the progression of CVD and T2D, as well as cognitive impairment, neurodegenerative disorders, and cancer [1,5,6].

CVD involves various heart conditions and blood vessel diseases, which include cerebrovascular disease, peripheral artery disease, valve disease, congenital heart disease, and thrombosis [7]. Recent findings indicate that endothelial dysfunction initiates atherosclerotic vascular disease and may serve as a predictor for future CVD events [8]. Vascular cell adhesion molecule-1 (VCAM-1) is a protein essential for leukocyte adhesion and transmigration to the interstitial space. This molecule has been suggested as a possible biomarker for CVD. During inflammation, macrophages, T lymphocytes, and natural killer cells release cytokines such as interleukin 1β (IL-1β) and tumor necrosis factor-α (TNFα). These cytokines activate the IκB kinase/nuclear factor kappa B (IKK/NF-kB) signaling pathway, resulting in increased VCAM-1 expression in various cells. VCAM-1 is converted into a soluble form, sVCAM-1, which is secreted into the interstitium and plasma [7]. Increased plasma levels of sVCAM-1 were observed in CVD [7] as well as in T2D [9]. Plasma sVCAM-1 levels are useful biomarkers for subclinical atherosclerosis, enabling early detection of at-risk, nonsmoking middle-aged individuals with MetS and guiding targeted primary prevention [6].

Diet and exercise have been suggested as the primary intervention strategies for the prevention of MetS. Mediterranean-style and vegetarian diets, characterized by the regular consumption of tree nuts, have been associated with a reduced risk of developing MetS [10]. Research indicates that nut consumption has the potential to reduce CMR factors and the incidence of CVD [11].

Walnut (*Juglans regia* L.), containing a balanced lipid profile and a significant concentration of bioactive antioxidant and anti-inflammatory compounds [12], has demonstrated potential health benefits [13]. Regular walnut consumption is linked to a reduced prevalence of CVD and T2D, healthy aging, as well as cancer prevention [14]. Walnuts are a significant source of unsaturated fatty acids and are also rich in fiber, minerals (potassium, calcium, and magnesium), vitamins (folate and vitamin E), phytosterols (campesterol, stigmasterol, and β-sitosterol), and polyphenols (ellagic acid (EA), gallic acid, and hydrolyzable tannins) [15]. They contain a high concentration of polyunsaturated fatty acids (PUFAS). Linoleic acid (18:2 *n*-6) and linolenic acid (18:3 *n*-3) predominate among these, with a ratio of around 4.2:1. The α-linolenic acid, an essential precursor of omega-3 fatty acids, possesses anti-inflammatory and antiatherogenic properties, which may prevent or have a beneficial impact on cardiometabolic and CVDs [16].

Dietary polyphenols offer various health benefits by influencing numerous physiological processes and counteracting oxidative stress (OS) and inflammation [17]. Recent studies demonstrate that ellagitannins (ETs) from walnuts can be converted to EA and subsequently be metabolized into urolithins by gut microbiota (GM). These compounds exhibit antioxidant and anti-inflammatory activity, cardiometabolic protection, and neuroprotection and could potentially modulate GM [15]. These effects should be considered when evaluating the health benefits of walnut consumption, particularly regarding the prevention of cardiometabolic risk and CVD, as well as the modulation of related health parameters.

Several clinical investigations have previously examined the impact of walnut consumption on sVCAM-1 [18,19,20,21,22] together with other selected cardiometabolic and anthropometric parameters [23,24,25,26,27,28]. These studies analyzed different parameters, such as the walnut amount, the control diet composition, the duration of walnut exposure, and participant demographics or their health status.

In a recent meta-analysis, we evaluated the impact of walnut consumption on biomarkers associated with MetS and inflammation in middle-aged and older adults. However, the statistical analysis of the endothelial adhesion molecule VCAM-1 was impeded by the limited number of studies that were selected for the assessment following the bias risk evaluation. Nevertheless, diets enriched with walnuts statistically significantly reduced the TG, TC, and LDL-c levels. In contrast, no significant changes were noted in anthropometric measurements or glycemic parameters [29]. The clinical data regarding the effects of walnut consumption on cardiometabolic risk parameters, especially on inflammatory and glycemic biomarkers, remain inconclusive in this age group.

The objective of our study was to conduct a cross-over randomized controlled trial (RCT) to examine the effects of daily short-term (4-week) walnut consumption on various cardiometabolic parameters in middle-aged adults at risk for MetS. We analyzed serum levels of sVCAM-1, lipid profiles, glucose metabolism, blood pressure, and various anthropometric measurements.

## 2. Materials and Methods

This study was performed following the CONSORT 2010 statement criteria, extended to include randomized crossover trials [30].

### 2.1. Study Design

The present research was designed as a crossover RCT to assess the effects of daily walnut consumption on sVCAM-1 levels and several cardiometabolic and anthropometric parameters. The trial consisted of two intervention periods of 28 days each, separated by a one-month (31 days) washout period to eliminate any carryover effects. Participants were randomly assigned to receive either a daily serving of walnut kernels (45 g per day each) or a habitual normocaloric diet without walnuts (control) in the first period, followed by the opposite treatment in the second period. This design allowed participants to serve as their control, enhancing the study’s statistical power.

Sample size estimation: We computed the sample size for the effect of the primary objective, sVCAM, to achieve 80% power with an alpha value of 0.05 and a two-tailed *p*-value. We based our estimation on data extracted from other studies [18,22], taking the largest mean difference and the largest standard deviation common to the two examples. Thus, we aimed to detect a difference in mean change (122.5 ng/mL), assuming a common standard deviation of 154.7. For this objective, we used GPower version 3.1.9.7 and found a sample size of 19 subjects.

### 2.2. Participants

The participants were recruited from the city of Cluj-Napoca, Romania, and were screened for eligibility based on the following criteria.

Inclusion Criteria: women and men aged 40–65 years, with no known allergy to walnuts, who provided informed consent. Clinical characteristics: individuals presenting at least one pathologically altered MetS-specific parameter, including (1) abdominal obesity, waist circumference (WC) ≥ 102 cm and ≥ 88 cm for men and women, respectively; (2) dyslipidemia, TG ≥ 150 mg/dL, and high-density lipoprotein cholesterol (HDL-c) < 40 mg/dL in men or < 50 mg/dL in women; (3) dysglycemia, fasting blood glucose (FBG) 100–125 mg/dL; and (4) hypertension, systolic blood pressure (SBP) ≥ 130 mmHg, or diastolic blood pressure (DBP) ≥ 85 mmHg [31].

Exclusion Criteria: individuals presenting (1) allergies to walnuts, other tree nuts, or peanuts, as well as those adhering to restrictive diets due to food allergies or intolerances (e.g., lactose, fructose, or gluten), diets associated with chronic gastrointestinal or kidney diseases, vegetarians, or any other dietary practices that could potentially affect study outcomes; (2) eating disorders within the six months preceding the study’s commencement were deemed ineligible; (3) chronic conditions: chronic intestinal diseases (e.g., ulcerative colitis and Crohn’s disease), chronic renal disease, CVD, pulmonary disease, type 1 or type 2 diabetes, cancer, neurodegenerative diseases (e.g., Alzheimer’s disease and Parkinson’s disease), and gallbladder disorders (e.g., gallbladder lithiasis, biliary dyskinesia, and acute or chronic cholecystitis). Additional exclusion criteria: pregnancy, smoking, chronic alcohol consumption, and the use of any medications or dietary supplements within a minimum of two weeks prior to the study’s initiation.

### 2.3. Interventions

The walnuts were harvested from orchards in Satu Mare County (47°47′24″ N, 22°53′24″ E), the northwestern part of Romania. The experimental protocol of this study is based on a daily intake of 45 g of walnuts integrated into a weight-maintenance diet.

Prior to enrollment in the study, all participants completed a food frequency questionnaire (Food4Me) [32,33] under the supervision of a registered dietitian. The daily caloric requirements for each participant were calculated using the Dietary Reference Intakes (DRI) calculator [34]. They received nutritional recommendations according to their individual energy requirements (1500 kcal, 2000 kcal, and 2500 kcal) to maintain their BW during the study and were also instructed to integrate walnuts into their diets during the intervention period. Subsequently, each person received general dietary recommendations based on current dietary guidelines, such as the Dietary Guidelines for Americans 2020–2025 [35] or the Food-Based Dietary Guidelines in Europe [36]. Moreover, the participants were advised to implement these recommendations at least two weeks prior to the baseline assessment and to continue following them throughout the three-month study. None of the participants adhered to a specific eating pattern or diet prior to the commencement of the study. All participants followed a habitual diet and showed no tendency to gain weight in the three months prior to enrollment. Caloric requirements and their distribution for maintaining body weight were calculated to take these characteristics into account.

Intervention group (*n* = 11): Participants consumed 45 g of fresh walnut kernels daily for 28 days as part of a normocaloric diet aligned with the broad nutritional recommendations of current dietary guidelines [36]. Additionally, it was considered that 45 g (approximately a small handful) is a practical and achievable amount for daily consumption, promoting adherence in real-world settings. This amount constituted approximately 15% of the total daily caloric intake for a standard 2000 kcal diet. It corresponds to roughly 1.5 servings of nuts, with a standard serving defined as 30 g. The calculation was based on the caloric density of nuts (654 kcal per 100 g), which yields about 294 kcal for a 45 g portion. Each participant received a total of 1260 g of walnut kernels, divided into 28 vacuum-packed packets of 45 g each, corresponding to the 28 days of treatment. Participants were instructed to store the walnuts away from light and at temperatures of 4 °C or lower. They were also advised to consume their walnut portion, if possible, in the earlier part of the day as a snack.

Control group (*n* = 9): Participants in the control group were instructed to adhere to a diet based on the broad nutritional recommendations outlined in current dietary guidelines [36] for maintaining body weight without any tree nuts, peanuts, or seed consumption for 28 days.

All study participants were advised to avoid the consumption of walnuts or other nuts, including tree nuts (almonds, Brazil nuts, cashews, hazelnuts, macadamia, pecans, pine nuts, pistachios), peanuts, and nut butters, along with seeds (chia, flax, pumpkin, sesame, sunflower, poppy, psyllium, and hemp) and seed butters (sesame or tahini and sunflower) throughout the study period, starting at least two weeks before the first assessment. Additionally, both groups (intervention and control) were advised to avoid dietary supplements with anti-inflammatory and antioxidant compounds, such as fish oil, omega-3 fatty acids, resveratrol, curcumin, vitamin C, selenium, and zinc, as well as dietary fiber, probiotics, and symbiotics, at least two weeks prior to the first test assessment and throughout the study.

Following the first period, when individuals in the intervention group consumed the daily walnut amount, there was a one-month (31-day) washout period to eliminate any potential side effects before they switched to the other intervention.

### 2.4. Randomization and Blinding

Participants were randomized into either the intervention or control group using a computer-generated randomization schedule. Five blocks of varying sizes—specifically, 2, 4, 6, and 8—were randomly created. A researcher managed the allocation process, distributing walnut packets according to the patient’s ID to ensure allocation concealment. Given the nature of the intervention, which involved walnut consumption, blinding participants was not possible. However, the laboratory technicians evaluated the outcomes, and the data analysts remained blind to the group assignments.

### 2.5. Data Collection and Outcome Measures

A total of four evaluations were performed, and the measured outcomes were assessed at the beginning and the end of each intervention period. The primary outcome was sVCAM-1, and the secondary outcomes included measurements of (1) anthropometric parameters—height, WC, hip circumference (HC), waist-to-hip circumference ratio (WHR), BW, body mass index (BMI), body fat mass (BFM), and body water—and (2) cardiometabolic parameters—SBP, DBP, TG, TC, HDL-c, LDL-c, FBG, and glycosylated hemoglobin A1c (HbA1c).

The parameters were selected based on their relevance to cardiovascular and metabolic health outcomes [1,2]. The main outcome, sVCAM-1, is a well-established biomarker of endothelial activation and inflammation in atherosclerosis [7]. Anthropometric parameters, including WC, BW, BMI, and BFM, are key indicators of body composition and obesity, which are linked to cardiometabolic and MetS risk. To evaluate metabolic health and heart disease risk, the SBP, DBP, TG, TC, HDL-c, LDL-c, FBG, and HbA1c levels were measured [1,2,3].

The clinical and paraclinical assessments were performed by a medical team from the Clinic of Occupational Medicine, Cluj County Emergency Clinical Hospital, Cluj-Napoca, Romania. For the data collection, standardized protocols were followed to ensure consistent and accurate measurements. Venous blood samples were collected (10 mL) in duplicate after an overnight fast, and the serum fractions were separated by centrifugation and analyzed to assess the cardiometabolic parameters and the biomarkers of oxidative stress and inflammation, respectively, using validated laboratory techniques.

#### 2.5.1. Anthropometric Measurements

The anthropometric measurements were performed in the morning after a fasting period of at least eight hours using a calibrated instrument (TANITA BC-545N Segmental Body Composition Scale, Tokyo, Japan; Gima taliometer, Milan, Italy). Participants were measured for their height and weight while standing in a barefoot position and wearing light clothes, using a stadiometer and an electronic digital scale. Height was recorded to the nearest 0.5 cm, and weight was measured to the nearest 0.1 kg. BMI was calculated by BW (kg) divided by height squared (m^2^). The WC was measured in a standing position with a relaxed abdomen and arms resting at the sides. The midpoint between the lower thoracic cage and iliac crest was recorded in the horizontal plane around the body. The WC was measured to the nearest 0.1 cm after a typical expiration [37,38]. The HC was determined at the widest position between the hips and buttocks. The WHR was determined by dividing the WC by the HC [39]. The definition of an increased WC was defined as a value ≥ 102 cm in men and ≥88 cm in women [40]. Additionally, we confirmed that none of the participants were in a state of acute illness or undergoing acute or subacute medical treatments on the day of each examination.

#### 2.5.2. sVCAM-1 Assessment

The serum levels of sVCAM-1 were evaluated using a commercially available ELISA kit (Human sVCAM-1 ELISA Kit, Invitrogen™, Vienna, Austria) in accordance with the manufacturer’s instructions. Microplate determinations were performed using a Synergy 2 Multi-Mode Microplate Reader. The results were expressed in ng/mL for VCAM.

#### 2.5.3. Cardiometabolic Parameters

Blood pressure (SBP and DBP) was measured at baseline and at the other scheduled clinic visits at one-month intervals using a professional automatic sphygmomanometer (GIMA TOKYO, Milan, Italy). Three blood pressure measurements were taken at three-minute intervals, and the average of the last two measurements was recorded.

Lipid profile assessments (TG, TC, LDL-c, and HDL-c) and glucose metabolism parameters (FBG and HbA1c) were performed by the nationally accredited Laboratory of the Clinic of Occupational Medicine, Cluj County Emergency Clinical Hospital, Cluj-Napoca, Romania.

### 2.6. Statistical Analysis

Data were analyzed using the R software environment for statistical computing and graphics (R Foundation for Statistical Computing, Vienna, Austria) version 4.3.2. Descriptive statistics were calculated for all variables, means and standard deviations for continuous ones, and absolute and relative frequencies for qualitative data. Linear mixed models were built with dependent variables represented by the change between the final and the baseline values for each variable. The independent variables were the intervention (walnut vs. control), the period (first and last), and the interaction term between the intervention and the period. The random effect was set for the patients. For each model, the coefficient, along with 95% confidence intervals and *p*-values, was reported. A *p*-value of <0.05 was considered statistically significant. Two-tailed *p*-values were considered for all statistical analyses.

### 2.7. Ethical Considerations

This study was conducted in accordance with the Declaration of Helsinki and was approved by the Scientific Research Ethics Committee of Iuliu Hatieganu University of Medicine and Pharmacy, Cluj-Napoca, Romania (AVZ 79/12 May 2023). All participants provided written informed consent before participation. Participant confidentiality was maintained throughout the study, and data were anonymized prior to analysis. Participants received no financial compensation. The trial was registered on ISCRTN with the clinical trial registration number ISRCTN17119161 and the date 26 September 2024.

## 3. Results

**Figure 1:**
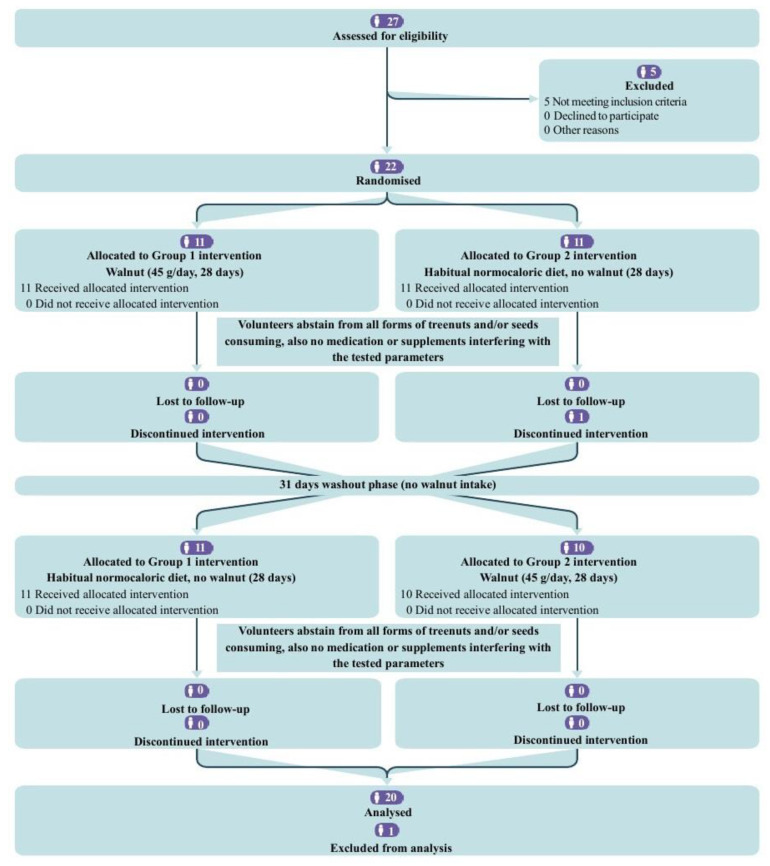
CONSORT flow diagram for the crossover trial.

In accordance with the inclusion criteria, 22 participants were enrolled in the study. Following the initial evaluation, one participant was required to resign from the study due to medical complications. One sample was excluded from analysis due to accidental deterioration. Consequently, 20 participants completed the study and were analyzed. The participants’ flow diagram is represented in Figure 1.

### 3.1. Baseline Characteristics

Table 1 and Supplementary Table S1 present the baseline characteristics by sequence and at the beginning of any of the two periods for all 20 subjects (47,67% female), with a mean age of 49.15 ± 4.11 years (values ranging from 42 to 56) (Table 1).

### 3.2. Nutrient Profile

Supplementary Table S2 presents the daily energy intakes and selected nutrients from diet example plans designed for the three energy categories of 1500, 2000, and 2500 kcal per day, respectively. The two diet types, one with 45 g of walnuts and the other without, are comparable in caloric intake and macronutrient ratios, such as total fat, net carbohydrate, and protein for each energy category. Due to the composition of walnuts, the interventional diet was distinguished by increased levels of copper, iron, magnesium, and zinc, as well as omega-3 and omega-6 PUFAs, in comparison to control diets where participants were advised to avoid nuts. Additionally, walnut diets contain reduced quantities of trans-lipids, saturated fats, and sodium.

### 3.3. Study Outcomes

The effect measure of interest was the change in characteristics represented by the difference between the final and baseline values (Table 2).

The walnut group had a significantly greater reduction in WC compared to the control group (*p* = 0.049). The differences in the rest of the anthropometric measurements (including hip circumference, BW, body fat mass, and body water percentage) and SBP or DBP did not reach statistical significance. While TG, TC, and low-LDL-c showed some reduction in the walnut group, none of these changes were statistically significant (*p*-values ranged from 0.123 to 0.208). Also, HDL-c did not show significant differences between the walnut and control groups, same as HbA1c changes. The difference between FBG levels showed a slight upward trend after walnut consumption but was not statistically significant (*p* = 0.089).

## 4. Discussion

The present crossover RCT evaluated the daily supplementation with 45 g of walnuts in the context of a weight maintenance diet over a period of 4 weeks in middle-aged participants in relatively good health but at risk of MetS. The effects of walnut consumption on several health parameters were assessed by comparing the changes from baseline between the walnut intervention group and the control group. A statistically significant decrease was observed between the two groups in waist circumference, with its decline after daily consumption of walnuts (*p* = 0.049), while the primary biomarker of interest, sVCAM-1, did not exhibit any significant differences among the other parameters that were examined.

The experimental protocol of our research, involving the ingestion of 45 g of walnuts daily, is consistent with the Food and Drug Administration’s (FDA) dietary guidelines, which recommend the inclusion of 1.5 ounces (42–43 g) of walnuts in the daily diet of adults [41]. Nevertheless, this quantity differs from the general dietary guidelines, which recommend consuming 30 g of nuts daily as part of a diversified and balanced diet [42].

The research protocol of our performed RTC was developed by applying the subgroup analysis data from a previous meta-analysis performed by our team. This protocol included variables such as participants’ health status, the amount of daily walnut consumption, diet type, and duration of walnut exposure. All subgroups assessed in the aforementioned meta-analysis reported statistically significant outcomes [29].

### 4.1. Anthropometric Profile

The outcomes indicated slight decreases in several parameters within the walnut intervention group compared to the control group (Table 2). These parameters included WC, hip circumference, body weight, BMI, as well as visceral and abdominal fat ratings. Notably, the WC showed a significant decrease (*p* = 0.049) in the walnut-treated group compared to the control group. This aspect is clinically relevant, as waist circumference serves as an important predictor of abdominal adiposity and metabolic health. Type 2 diabetes, cardiovascular disease, and metabolic syndrome are all associated with abdominal adiposity, and the inclusion of walnuts in the daily diet may mitigate these risks by promoting a healthier waist circumference [43].

Our results, obtained after a short 4-week intervention period, are in line with the findings of Estruch et al. in the PREDIMED (“Prevención con Dieta Mediterránea”) 5-year parallel RCT, which involved 7447 participants with T2D or at high risk of CVD.

It is important to note that the results should be interpreted within the context of various dietary modifications [43]. Additionally, a significant association was found between lower WC and walnut consumption in the long-term Coronary Artery Risk Development in Young Adults (CARDIA) study [44]. Furthermore, the findings from the 2-year parallel RCT conducted by Abdrabalnabi et al. [10] as part of the Walnuts and Healthy Aging (WAHA) research involving 625 healthy older adults with an average age of 69.1 years, indicated that consuming walnuts as ~15% of their total energy needs did not significantly alter their BMI. Similarly, a recent short-term study by Soares et al. [28] over a period of 45 days and involving 24 healthy participants with an average age of 36.8 years also showed no statistically significant changes in BMI following a daily intake of 24 g of walnuts.

However, the anthropometric parameters provided statistically significant results in numerous walnut-related studies. Rock et al. observed that a reduced energy-density walnut diet resulted in significant reductions in BW, BMI, and WC in 100 healthy, overweight, and obese volunteers [45]. The 2-year parallel RCT conducted by Bitock et al. in the context of WAHA study resulted in a decrease in BW (*p* = 0.031), an increase in BF (*p* = 0.0001), and no change in WC or WHR in 183 healthy elderly participants (mean age of 69 years) after consuming 28–56 g of walnuts per day [46]. A recent small-scale randomized study on 18 healthy people that consumed 56 g of walnuts for a period of four weeks showed a statistically significant decrease in BMI (*p* = 0.0064) [47].

The current RCT results are also in agreement with the most recent meta-analyses that evaluated anthropometric parameters after nut consumption. Meta-regressions demonstrated that higher nut consumption correlated with reductions in BW and body fat mass, thereby alleviating concerns regarding the potential for nuts to contribute to increased adiposity [48,49]. Several meta-analyses showed that measurements like BW, BMI, body fat mass, and WC did not significantly differ from the control group after consuming diets rich in walnuts [29,50].

This study found no statistically significant changes in body weight or fat mass as a result of walnut consumption. Consequently, extended intervention durations might provide a more profound understanding of the impacts on body composition. Long-term studies could be helpful in figuring out whether consuming walnuts regularly causes greater improvements and, more importantly, if these changes promote metabolic health.

### 4.2. sVCAM-1

Our research indicates that short-term (4-week) dietary supplementation with 45 g of walnuts per day, as part of a body weight maintenance diet, does not significantly reduce sVCAM-1 levels in the selected population.

The findings correspond with those reported by Damasceno et al., who performed a 4-week crossover RCT with 18 overweight hypercholesterolemic patients (mean age 56 ± 13 years) consuming 40–65 g of walnuts daily [20]. Furthermore, Cofan et al. performed a 2-year parallel RCT with 634 healthy participants (average age: 69.1 ± 3.6 years) who consumed 30–60 g of walnuts per day [19]. Both studies reported no significant changes in sVCAM levels, indicating that the effects of walnut consumption may vary based on individual health status or dietary context. In addition, the systematic review and meta-analysis by Hsu et al. found no significant decrease in VCAM-1 levels related to walnut consumption’s impact on markers of endothelial function in adults but indicated that walnuts may minimize the CVD risk by increasing flow-mediated dilation (FMD) [51].

Several previous studies demonstrate that acute and chronic walnut intake can significantly reduce sVCAM levels, suggesting potential cardiovascular benefits. For instance, both Cortes et al. [52] and Bhardwaj et al. [8] reported significant decreases in sVCAM following an acute walnut intake of 40 g by healthy overweight volunteers and 60 g by hypercholesterolemic patients, respectively, compared to control. Furthermore, the crossover design randomized trials performed by Ros et al. [22] and Canales et al. [18] provide compelling evidence regarding the impact of walnut consumption on endothelial function, as indicated by sVCAM levels. In the first study, an administration of 40–65 g of walnuts per day vs. a cholesterol-lowering isocaloric Mediterranean diet resulted in a statistically significant reduction (*p* = 0.045) in sVCAM levels among 20 hypercholesterolemic volunteers (55 ± 5.9 years) after four weeks [22]. Similarly, the second study revealed that participants (*n* = 22, 54.8 ± 9.4 years) at increased cardiovascular risk who consumed walnut-enriched meat products (approx. 22 g walnuts/day) exhibited significantly lower sVCAM levels (*p* = 0.012) compared to those consuming low-fat meat products [18].

All the findings illustrate the intricacies of dietary interventions in cardiovascular health and highlight the necessity for additional research to elucidate the underlying mechanisms and validate these results across varied populations.

### 4.3. Blood Pressure

The current investigation revealed a slight increase in SBP, while DBP decreased in the intervention group compared to the control, although the obtained values were not statistically significant. Several previous studies revealed lower SBP levels following walnut consumption, but mostly without statistical significance [10,45,47,53]. However, the DBP decrease noticed in our research confirms several earlier results [10,45,47,53,54]. Notably, of the mentioned studies, only one [54] was designed as a randomized controlled crossover trial, with most of them conceived as parallel trials. Rock et al. showed reductions in both SBP and DBP. In contrast to our investigation, these changes were accomplished within the context of a behavioral weight loss intervention targeting overweight and obese participants [45]. Our results are mainly comparable to those of a crossover RCT that involved 45 participants (mean age 43 ± 10 years) at risk of CVD. That study demonstrated a reduction in DBP compared to the control group (*p* = 0.051), whereas SBP remained unchanged. These results were obtained after following three distinct isocaloric weight maintenance diets over a period of 6 weeks. [54].

The data regarding SBP diverged from findings in the two-year WAHA substudy, which examined the impact of a walnut (30–60 g per day) diet on blood pressure among elderly participants (*n* = 236, mean age 69 years, 60% with mild hypertension). Compared to the control group, the average 24 h ambulatory SBP showed a significant change (*p* = 0.034) only in the walnut group of participants who began with a baseline SBP over 125 mmHg. Moreover, they did not observe any alterations in DBP [55]. In a separate WAHA study, Abdrabalnabi et al. found that walnut supplementation diets did not significantly affect any of the individual components of MetS among 625 participants (mean age: 69.1 years) when compared to the control group, which aligns with our findings. However, the walnut group exhibited a greater reduction in both SBP and DBP compared to the control group [10]. Statistically significant reductions in SBP (*p* = 0.0015) as well as in DBP (*p* = 0.0793) were noted in the small-scale randomized study conducted by Petrovic-Oggiano et al. [47]. However, Soares et al. were unable to validate the results, perhaps due to the insufficient quantity of walnuts and the brief duration of the intervention [28].

The results of our study in terms of blood pressure changes concur with several meta-analyses of RCTs meeting the criteria of our trial, where both SBP and DBP did not show statistically significantly changed outcomes [29,56,57].

### 4.4. Lipid Profile

One significant modifiable risk factor for MetS is dyslipidemia, characterized by elevated plasma levels of either TC, LDL-c, or TG or decreased plasma levels of HDL-c [58]. Within the tree nut family, walnuts have been previously found to have cholesterol-lowering and cardioprotective effects [59]. Our study indicates a trend in the reductions in TG, TC, and LDL-c levels, as well as elevations in HDL-c levels, compared to the control group. Additionally, the lipid profile values of the control group increased in response to the standard diet, whereas they decreased in the group that received walnuts, demonstrating disparities between the two groups. In contrast, HDL-c levels show a beneficial increase in both groups, with the walnut group experiencing a greater increase.

These results are consistent with earlier evidence suggesting that walnut consumption may improve the lipid profile. In this regard, total cholesterol exhibited a decreasing tendency, although fasting LDL-c, HDL-c, and triglycerides did not show significant changes in an eight-week crossover randomized controlled trial including healthy Caucasian participants (*n* = 40, mean age 60 ± 1 year) after a daily intake of 43 g of walnuts in comparison to the control diet [21]. Likewise, another crossover RCT indicated unchanged levels of triglycerides and total cholesterol in 90 healthy volunteers (mean age 54.3 years) after a walnut-supplemented diet (12% of energy consumption) over a 6-month period [60].

Nevertheless, several statistically significant results have been reported, both in parallel and in crossover trials. In a cross-over RCT including 194 healthy Caucasian participants (mean age 63 ± 7 years) that followed a walnut-enriched diet (43 g per day) for 8 weeks, a significant reduction in TC (*p* = 0.002), LDL-c (*p* = 0.029), and TG (*p* = 0.015) was noticed, while HDL-c did not change significantly [61]. In addition, TC and LDL-c significantly decreased after 6 months of a walnut-enriched hypocaloric diet in healthy, overweight, and obese volunteers [45]. Moreover, in a crossover RCT that enrolled 119 Korean adults with MetS, HDL-c significantly improved (*p* = 0.028) following 45 g of walnut intake for 16 weeks [23]. In the context of the WAHA study, the walnut diet significantly decreased TC and LDL-c, whereas TG and HDL-c were unaffected in elderly volunteers without major comorbidities (*n* = 708, mean age 69 years) following a walnut-supplemented diet (30–60 g per day) [27]. Another substudy of WAHA reported a 1-year decrease in LDL-c (*p* = 0.010) vs. the control group [62].

The meta-analysis evaluating various biomarkers of MetS and inflammation following walnut intake interventions in individuals over 40 years demonstrated a significant enhancement in lipid profiles (TG, TC, and LDL-c levels) associated with walnut consumption. However, there was significant variability in the intervention methodologies compared to the various control diets [29]. Moreover, these results corroborated the conclusions of earlier meta-analyses [63,64].

### 4.5. Glycemic Profile

Regarding glycemic profile, neither of the two tested parameters showed significant decreases after nut consumption. FBG levels even registered a slight increase in the walnut intervention group when compared with the control diet group.

These results confirm those obtained in several previous investigations. For example, both FBG and HbA1c did not change significantly in a crossover RCT when the walnut group was compared with a Western diet [21]. Similarly, in the two-year parallel RCT (WAHA) performed by Abdrabalnabi et al., FBG decreased in both the walnut and the habitual diet groups, but no significant differences between the groups were observed in the development or reversion of MetS [10].

However, other studies showed significant improvements in both FBG (*p* = 0.013) and HbA1c (*p* = 0.021) levels after walnut intake [23]. Furthermore, FBG significantly decreased (*p* < 0.02) after consuming a smoothie containing 48 g of walnuts compared with the control in a crossover RCT that involved 10 obese volunteers [24]. Likewise, statistically significant results for FBG (*p* = 0.01) have been obtained in a very recent clinical trial on healthy volunteers (*n* = 24, mean age 36.8 years) after 25 g of walnut kernel daily intake over a period of 45 days [28].

The displacement of high-carbohydrate foods has been identified as a consequence of the inclusion of walnuts in the diets of individuals at risk for or with T2D. A mechanistic explanation might be that the dietary consumption of monounsaturated fatty acids and PUFAs in place of carbohydrates can enhance insulin sensitivity and lower insulin secretion and blood glucose levels [65]. Nevertheless, the reported effects of walnut consumption on glycemic control in T2D are still inconclusive.

## 5. Strengths, Limitations, and Future Prospects

This study has several strengths that enhance the validity and reliability of its findings. First, and most importantly, the crossover design allowed each participant to serve as their control, reducing the impact of inter-individual variability and increasing the power of the study to detect differences between the walnut and control interventions. Additionally, the implementation of a one-month washout period between intervention phases minimized the potential carryover effects.

Despite the rigorous design of this randomized controlled crossover trial, it is important to acknowledge some limitations. First, the inability to blind participants due to the intervention may have introduced bias, as participants’ awareness of their group assignment could influence their eating behavior and lifestyle. Second, the relatively short duration of the intervention periods may have been insufficient to achieve significant changes in some cardiometabolic and anthropometric outcomes. Additionally, the study’s sample size was relatively small, which may limit the statistical power to detect subtle differences between the intervention groups. The slight difference in the number of women between the two groups might have affected the results, considering the biological variances, such as hormone changes, body composition, and metabolism. The investigation included individuals over the age of 40 who exhibited at least one specific parameter of the MetS with pathological values but no drug treatment. This characteristic resulted in a significant degree of variability among the study participants, and the limited number of participants rendered it impossible to divide them and assess them by subgroups. Moreover, we believe that the relatively good health status of these participants minimized discrepancies in the tested parameters, which might have otherwise allowed for a significant change to be observed within the four-week period. In this study, all participants consumed the same type of walnut. But the differences in walnut composition—such as their content of healthy fats, antioxidants, fibers, and phytochemicals, which are influenced by species-specific characteristics, soil composition, geographic factors, and climatic conditions—could influence the extrapolation of the results obtained in this study to other populations. Furthermore, the absence of phytochemical analysis of the walnut samples, coupled with the lack of standardization in walnut composition, makes it difficult to compare our outcomes with other results.

In the scientific literature, there are relatively few RCT studies that have specifically investigated the impact of daily walnut consumption on middle-aged subjects, and the results are contradictory. Due to its rigor, this RCT could be considered valuable, and the obtained results may contribute to consolidating the knowledge in the field through their inclusion in meta-analyses.

## 6. Conclusions

In the present randomized, controlled, crossover trial, the daily consumption of 45 g of walnuts within the context of a weight-maintenance diet over four weeks resulted in a statistically significant reduction in waist circumference. However, there were no statistically significant changes in sVCAM-1, selected cardiometabolic parameters, or the anthropometric parameters observed among a cohort of middle-aged, generally healthy Caucasian individuals at risk for MetS. These findings highlight the need for longer-term studies to better understand the potential benefits of walnut supplementation in preventing and managing MetS within this demographic category.

## Figures and Tables

**Table 1:** Baseline characteristics of participants by sequence.

Variables	Control–Walnut (*n* = 9)	Walnut–Control (*n* = 11)
Age (years)	47.67 (3.28)	49 (4.22)
Female, *n* (%)	4 (44.44)	6 (54.55)
Height (cm)	173.78 (5.87)	171 (8.04)
Waist circumference (cm)	102 (5.66)	96 (7.16)
Hip circumference (cm)	109.33 (7.42)	107.55 (4.52)
Waist–hip ratio	0.93 (0.05)	0.89 (0.07)
Body weight (kg)	91.81 (11.86)	82.33 (10.92)
BMI (kg/m^2^)	30.02 (2.85)	28.14 (2.73)
Body fat mass (%)	30.73 (9.36)	31.55 (9)
Body water (%)	50.32 (5.93)	49.8 (5.86)
VAFR	10.22 (2.22)	9.18 (2.26)
s-VCAM-1 (ng/mL)	968.25 (209.13)	796.78 (228.25)
SBP (mmHg)	127.22 (10.93)	115.45 (9.34)
DBP (mmHg)	83.33 (9.01)	73.18 (7.83)
TG (mg/dL)	138.33 (56.72)	115 (37.38)
TC (mg/dL)	197.44 (21.78)	233.91 (49.61)
LDL-c (mg/dL)	120.33 (22.39)	148.73 (38.78)
HDL-c (mg/dL)	49.44 (8.25)	62.18 (10.88)
FBG (mg/dL)	90.67 (6.78)	85.73 (8.51)
HbA1c (%)	5.6 (0.25)	5.66 (0.28)

Data are expressed as mean (SD), except for qualitative variables, which are expressed as *n* (%). BMI—body mass index; DBP—diastolic blood pressure; FBG—fasting blood glucose; HbA1c—glycosylated hemoglobin A1c; HDL-c—high-density lipoprotein cholesterol; LDL-c—low-density lipoprotein cholesterol; s-VCAM-1—soluble vascular cell adhesion molecule-1; SBP—systolic blood pressure; SD—standard deviation; TC—total cholesterol; TG—triglycerides; VAFR—visceral and abdominal fat rating.

**Table 2:** Change (final—baseline) in participant outcomes in walnut and control groups.

Variables	Control (*n* = 20)	Walnut (*n* = 20)	Difference LS (95% CI)	*p*-Value
Waist circumference (cm)	−0.7 (1.53)	−1.45 (1.19)	−1.22 (−2.44–−0.01)	0.049
Hip circumference (cm)	−0.4 (1.35)	−0.6 (0.99)	−0.73 (−1.81–0.36)	0.181
Waist–hip ratio	0 (0.01)	−0.01 (0.01)	−0.01 (−0.02–0.01)	0.308
Body weight (kg)	−0.44 (1.69)	−0.52 (1.18)	−0.72 (−2.04–0.6)	0.275
BMI (kg/m^2^)	−0.16 (0.56)	−0.19 (0.4)	−0.26 (−0.7–0.18)	0.235
Body fat mass (%)	−0.42 (1.69)	0.05 (1.05)	0.05 (−1.23–1.34)	0.933
Body water (%)	0.11 (1.39)	−0.09 (0.87)	0.31 (−0.73–1.35)	0.551
VAFR	−0.1 (0.52)	−0.15 (0.46)	−0.15 (−0.6–0.3)	0.513
s-VCAM-1 (ng/mL)	−46.54 (98.06)	−38.63 (147.84)	2.87 (−114.24–119.98)	0.961
SBP (mmHg)	1.5 (10.4)	−2.65 (10.58)	−1.21 (−10.78–8.35)	0.798
DBP (mmHg)	2.75 (11.18)	0.75 (7.83)	−1.62 (−10.57–7.34)	0.716
TG (mg/dL)	12.3 (46.95)	−6.3 (43.47)	−32.12 (−74.09–9.85)	0.129
TC (mg/dL)	10.15 (21.83)	−1.65 (28.3)	−18.26 (−41.73–5.2)	0.123
LDL-c (mg/dL)	6.1 (15.66)	−3.6 (24.82)	−12.16 (−31.42–7.1)	0.208
HDL-c (mg/dL)	1.65 (4.97)	3.3 (6.63)	0.37 (−5–5.75)	0.888
FBG (mg/dL)	−2.2 (5.75)	2.45 (6.83)	4.24 (−0.68–9.17)	0.089
HbA1c (%)	−0.12 (0.17)	−0.06 (0.12)	0.01 (−0.12–0.13)	0.933

Data are expressed as mean (SD) change (difference between the final and the baseline values) for all quantitative variables. The difference column presents the difference between treatment changes for the control and walnut groups obtained by linear mixed models. The linear mixed models were fit to predict change (difference between final and baseline values) in outcome variables, in function of the treatment, adjusted for period, with interactions, and with random effects for patients. BMI—body mass index; CI—confidence interval; DBP—diastolic blood pressure; FBG—fasting blood glucose; HbA1c—glycosylated hemoglobin A1c; HDL-c—high-density lipoprotein cholesterol; LDL-c—low-density lipoprotein cholesterol; LS—least squares; s-VCAM-1—soluble vascular cell adhesion molecule-1; SBP—systolic blood pressure; SD—standard deviation; TC—total cholesterol; TG—triglycerides; VAFR—visceral and abdominal fat rating.

## Data Availability

The original contributions presented in this study are included in the article. Further inquiries can be directed to the corresponding author.

## Supplementary Materials

The following supporting information can be downloaded at: https://www.mdpi.com/article/10.3390/nu17132072/s1. Table S1: Baseline characteristics for all subjects at the beginning of any of the two periods; Table S2: Daily energy intakes and selected nutrients from the diet example plans; Table S3: Final characteristics for all subjects at the end of any of the two periods; Table S4: Unadjusted change (final—baseline) in participants outcomes in walnut and control groups; Table S5: Linear mixed models predicting change (difference between final and baseline values) in outcome variables, in function of the treatment, adjusted for period, with interactions, and with random effects for patients. File S1. CONSORT 2010 checklist of information to include when reporting a randomised trial. (References [66,67] are cited in the supplementary materials).

## Abbreviations

Apo B—apolipoprotein B; BMI—body mass index; BW—body weight; CMR—cardiometabolic risk; CVD—cardiovascular disease; DBP—diastolic blood pressure; EA—ellagic acid; ETs—ellagitannins; FBG—fasting blood glucose; HbA1c—glycosylated hemoglobin A1c; HDL-c—high-density lipoprotein cholesterol; HOMA-IR—homeostatic model assessment for insulin resistance; ICAM-1—intracellular adhesion molecule-1; IKK—IκB kinase; IL-1β—interleukin 1β; IL-6—interleukin-6; INF-γ—interferon-gamma; IκB—inhibitor of κB; LDL-c—low-density lipoprotein cholesterol; MDA—malondialdehyde; MetS—metabolic syndrome; NF-kB—nuclear factor kappa B; ORAC—oxygen radical absorbance capacity; PUFAs—polyunsaturated fatty acids; RCT—randomized controlled trial; ROS—reactive oxygen species; SBP—systolic blood pressure; SCFA—short-chain fatty acids; SD—standard deviation; s-VCAM-1—soluble vascular cell adhesion molecule-1; T2D—type 2 diabetes; TC—total cholesterol; TG—triglycerides; TNFα—tumor necrosis factor-α; VAFR—visceral and abdominal fat rating; VCAM-1—vascular cellular adhesion molecule-1; WC—waist circumference; WHR—waist-to-hip circumference ratio.
