# Supplementary material for: Benefits of Short-Term (4-Week) Daily Walnut Consumption in Middle-Aged Adults at Risk for Metabolic Syndrome: Outcomes of a Randomized Controlled Trial
Source: Nutrients. 2025 Jun 21;17(13):2072. doi: 10.3390/nu17132072 (PMC12250811; doi:10.3390/nu17132072)
Supplement: Supplementary file 1 [file nutrients-17-02072-s001.zip › Supplementary file_nutrients 3704381_Revised_20.06.2025.pdf]

*Supplementary File*

# Benefits of Short-Term (4-Week) Daily Walnut Consumption in Middle-Aged Adults at Risk for Metabolic Syndrome: Outcomes of a Randomized Controlled Trial

**Letiția Mateș [1], Doina Albert-Ani [2], Ionel Fizeșan [1], Andreea-Elena Petru [1], Roxana Banc [3,*], Marius Emil Rusu [4], Carmen Costache [5,6], Lorena Filip [3,7], Daniela-Saveta Popa [1,†] and Daniel-Corneliu Leucuța [8,†]**

[1] Department of Toxicology, Faculty of Pharmacy, Iuliu Hatieganu University of Medicine and Pharmacy, 8 Victor Babes, 400012 Cluj-Napoca, Romania; micu.letitia@umfcluj.ro (L.M.); ionel.fizesan@umfcluj.ro (I.F.); petruandreeaelena@gmail.com (A.-E.P.); dpopa@umfcluj.ro (D.-S.P.)
[2] Clinic of Occupational Medicine, Cluj County Emergency Clinical Hospital, 3-5 Clinicilor Street, 400006 Cluj-Napoca, Romania; doinapirvalbert@gmail.com
[3] Department of Bromatology, Hygiene, Nutrition, Faculty of Pharmacy, Iuliu Hatieganu University of Medicine and Pharmacy, 6 Pasteur Street, 400349 Cluj-Napoca, Romania; lfilip@umfcluj.ro
[4] Department of Pharmaceutical Technology and Biopharmaceutics, Faculty of Pharmacy, Iuliu Hatieganu University of Medicine and Pharmacy, 8 Victor Babes, 400012 Cluj-Napoca, Romania; rusu.marius@umfcluj.ro
[5] Department of Microbiology, Faculty of Medicine, Iuliu Hatieganu University of Medicine and Pharmacy, 400012 Cluj-Napoca, Romania; anca.costache@umfcluj.ro
[6] County Emergency Hospital Cluj-Napoca, 3-5 Clinicilor Street, 400006 Cluj-Napoca, Romania
[7] Academy of Romanian Scientists (AOSR), 3 Ilfov Street, 050044 Bucharest, Romania
[8] Department of Medical Informatics and Biostatistics, Faculty of Medicine, Iuliu Hatieganu University of Medicine and Pharmacy, 6 Pasteur Street, 400012 Cluj-Napoca, Romania; dleucuta@umfcluj.ro
* Correspondence: roxana.banc@umfcluj.ro; Tel.: +40-744-367-958
† These authors contributed equally to this work.

**Table S1.** Baseline characteristics for all subjects at the beginning of the two intervention periods.

| Variables | Control (n=20) | Walnut (n=20) | P-value |
|---|---|---|---|
| Waist circumference (cm) | 97.35 (7.79) | 98 (6.8) | 0.780 |
| Hip circumference (cm) | 107.8 (6.01) | 108.3 (5.96) | 0.793 |
| Waist-hip ratio | 0.9 (0.06) | 0.9 (0.06) | 0.898 |
| Body weight (kg) | 86.17 (12.49) | 86.66 (12.15) | 0.90 |
| BMI ($kg/m^2$) | 28.82 (2.93) | 29 (2.89) | 0.838 |
| Body fat mass (%) | 31.22 (8.8) | 30.99 (8.87) | 0.936 |
| Body water (%) | 49.97 (5.65) | 50.19 (5.69) | 0.905 |
| Visceral/abdominal fat rating | 9.65 (2.36) | 9.62 (2.19) | 0.967 |
| s-VCAM-1 (ng/mL) | 788.32 (272.48) | 798.84 (204.89) | 0.891 |
| SBP (mmHg) | 121.25 (11.46) | 119.5 (9.99) | 0.610 |
| DBP (mmHg) | 77.25 (9.52) | 78.5 (9.61) | 0.682 |
| TG (mg/dL) | 121.4 (49.19) | 133.1 (50.41) | 0.462 |
| TC (mg/dL) | 208.85 (29.69) | 219.45 (45.62) | 0.389 |
| LDL-c (mg/dL) | 128.15 (26.59) | 136 (35.77) | 0.436 |
| HDL-c (mg/dL) | 56.4 (9.74) | 56.85 (11.08) | 0.892 |
| FBG (mg/dL) | 87.2 (7.78) | 86.5 (7.32) | 0.771 |
| HbA1c (%) | 5.67 (0.24) | 5.66 (0.26) | 0.915 |

Data are expressed as mean (SD) for all quantitative variables.
BMI – body mass index; DBP – diastolic blood pressure; FBG – fasting blood glucose; HbA1c – glycosylated hemoglobin A1c; HDL-c – high-density lipoprotein cholesterol; LDL-c – low-density lipoprotein cholesterol; SBP – systolic blood pressure; SD – standard deviation; s-VCAM-1 – soluble vascular cell adhesion molecule-1; TC – total cholesterol; TG – triglycerides.

**Table S2.** Daily energy intakes and selected nutrients from the diet example plans.

| | Diet type | | | | | |
|---|---|---|---|---|---|---|
| | **1500 kcal** no walnut | **1500 kcal** walnut | **2000 kcal** no walnut | **2000 kcal** walnut | **2500 kcal** no walnut | **2500 kcal** walnut |
| Participants | n=7 | n=7 | n=8 | n=8 | n=7 | n=7 |
| **Energy**, kcal | 1531 | 1536 | 2015 | 2005 | 2506 | 2537 |
| **Total protein** (≈ 25% TE), g/d | 92.4 | 92.9 | 124.7 | 126 | 147.7 | 152.3 |
| Total carbohydrates, g/d | 161.1 | 157.5 | 215.9 | 213.6 | 293.8 | 296.7 |
| **Net carbohydrates** (≈ 45% TE), g/d | 140 | 133.7 | 192.3 | 187.2 | 260.3 | 260.2 |
| Fiber, g/d | 19.3 | 21.9 | 21.7 | 24.4 | 31.3 | 34.2 |
| **Total fat** (≈ 30% TE), g/d | 59 | 62.9 | 73.4 | 74.5 | 84 | 85.9 |
| MUFA, g/d | 19.6 | 16.5 | 23.5 | 19.5 | 28.6 | 24.8 |
| PUFA, g/d | 8.9 | 29 | 11.5 | 31.6 | 15.4 | 35.5 |
| *ω-3* PUFA, g/d | 1 | 4.9 | 1.4 | 5.2 | 1.7 | 5.6 |
| *ω-6* PUFA, g/d | 6.8 | 23.6 | 8.9 | 25.6 | 12.2 | 29 |
| SFA, g/d | 24.2 | 11.4 | 30.4 | 15.9 | 31.1 | 16.9 |
| Trans-fats, g/d | 1.1 | 0.2 | 1.5 | 0.4 | 1.4 | 0.3 |
| Vitamin B1, mg/d | 1.4 | 1.4 | 1.8 | 1.8 | 2.5 | 2.6 |
| Vitamin B6, mg/d | 2.4 | 2.6 | 3.2 | 3.4 | 4.2 | 4.4 |
| Vitamin B12, μg/d | 2.9 | 2 | 3.8 | 3 | 3.4 | 3.1 |
| Folate, μg/d | 373 | 406.7 | 461.3 | 496.2 | 684.1 | 724 |
| Vitamin C, mg/d | 95.2 | 95.7 | 107.2 | 107.7 | 155.6 | 156.1 |
| Vitamin D, IU/d | 95.9 | 87.8 | 102.2 | 94.4 | 101.7 | 96.6 |
| Vitamin E, mg/d | 6.6 | 6.2 | 7.4 | 7 | 10.1 | 9.6 |
| Vitamin K, μg/d | 172.6 | 171.5 | 185.7 | 184.4 | 325.9 | 324.7 |
| Calcium, mg/d | 603.1 | 411.1 | 756.3 | 593.9 | 746.1 | 705.9 |
| Copper, mg/d | 0.8 | 1.5 | 1 | 1.7 | 1.4 | 2.1 |
| Iron, mg/d | 10.8 | 12 | 14.2 | 15.5 | 20 | 21.3 |
| Magnesium, mg/d | 227.1 | 278.4 | 288.5 | 342.3 | 375.7 | 439.7 |
| Phosphorus (mg) | 1151.2 | 1138.4 | 1479.9 | 1487.8 | 1729.3 | 1823.2 |
| Potassium (mg) | 2617.8 | 2507.5 | 3075.7 | 3004.8 | 3789.4 | 3876.3 |
| Selenium (μg) | 118.4 | 113.4 | 156.5 | 152.3 | 191.5 | 191.1 |
| Sodium, mg/d | 1749.3 | 1649.1 | 2319.4 | 2231.8 | 2577.1 | 2341.7 |
| Zinc, mg/d | 7 | 7.6 | 9.2 | 9.9 | 11.2 | 12.3 |

% TE – percentage from total energy (kcal); MUFA – monounsaturated fatty acids; PUFA – polyunsaturated fatty acids; SFA – saturated fatty acids.

Cronometer. (2024). *Cronometer (Version 4.0.0) [Desktop app]*. Spurrell Technology Inc. https://www.cronometer.com (accessed on 14 August 2023).

**Table S3**. Final characteristics for all subjects at the end of the two intervention periods.

| **Variables** | **Control (n=20)** | **Walnut (n=20)** |
|---|---|---|
| Waist circumference (cm) | 96.65 (7.6) | 96.55 (7.07) |
| Hip circumference (cm) | 107.4 (6.05) | 107.7 (5.9) |
| Waist-hip ratio | 0.9 (0.06) | 0.89 (0.06) |
| Body weight (kg) | 85.73 (12.58) | 86.14 (12.19) |
| BMI (kg/m$^2$) | 28.65 (3.03) | 28.82 (2.84) |
| Body fat mass (%) | 30.79 (8.13) | 31.04 (8.52) |
| Body water (%) | 50.08 (5.32) | 50.1 (5.53) |
| Visceral/abdominal fat rating | 9.55 (2.44) | 9.47 (2.22) |
| s-VCAM-1 (ng/mL) | 741.78 (262.73) | 760.21 (166.68) |
| SBP (mmHg) | 122.75 (12.51) | 116.85 (11.68) |
| DBP (mmHg) | 80 (12.57) | 79.25 (8.47) |
| TG (mg/dL) | 133.7 (56.91) | 126.8 (51.35) |
| TC (mg/dL) | 219 (34.98) | 217.8 (38.36) |
| LDL-c (mg/dL) | 134.25 (32.33) | 132.4 (33.89) |
| HDL-c (mg/dL) | 58.05 (9.28) | 60.15 (9.57) |
| FBG (mg/dL) | 85 (7.91) | 88.95 (10.39) |
| HbA1c (%), | 5.54 (0.19) | 5.6 (0.2) |

Data are expressed as mean (SD) for all quantitative variables.
BMI – body mass index; DBP – diastolic blood pressure; FBG – fasting blood glucose; HbA1c – glycosylated hemoglobin A1c; HDL-c – high-density lipoprotein cholesterol; LDL-c – low-density lipoprotein cholesterol; SBP – systolic blood pressure; SD – standard deviation; s-VCAM-1 – soluble vascular cell adhesion molecule-1; TC – total cholesterol; TG – triglycerides.

**Table S4.** Unadjusted change (final – baseline) in participants outcomes in walnut and control groups.

| **Variables** | **Control (n=20)** | **Walnut (n=20)** | **Difference Control-Walnut (95% CI)** |
|---|---|---|---|
| Waist circumference (cm) | -0.7 (1.53) | -1.45 (1.19) | 0.75 (-0.13 - 1.63) |
| Hip circumference (cm) | -0.4 (1.35) | -0.6 (0.99) | 0.2 (-0.56 - 0.96) |
| Waist-hip ratio | 0 (0.01) | -0.01 (0.01) | 0 (0 - 0.01) |
| Weight (kg) | -0.44 (1.69) | -0.52 (1.18) | 0.08 (-0.86 - 1.01) |
| BMI (kg/m$^2$) | -0.16 (0.56) | -0.19 (0.4) | 0.03 (-0.29 - 0.34) |
| Body fat mass (%) | -0.42 (1.69) | 0.05 (1.05) | 0.47 (-0.43 - 1.37) |
| Body water (%) | 0.11 (1.39) | -0.09 (0.87) | 0.2 (-0.55 - 0.94) |
| Visceral/abdominal fat rating | -0.1 (0.52) | -0.15 (0.46) | 0.04 (-0.27 - 0.36) |
| s-VCAM-1 (ng/mL) | -46.54 (98.06) | -38.63 (147.84) | 7.91 (-72.4 - 88.22) |
| SBP (mmHg) | 1.5 (10.4) | -2.65 (10.58) | 4.15 (-2.57 - 10.87) |
| DBP (mmHg) | 2.75 (11.18) | 0.75 (7.83) | 2 (-4.18 - 8.18) |
| TG (mg/dL) | 12.3 (46.95) | -6.3 (43.47) | 18.6 (-10.37 - 47.57) |
| TC (mg/dL) | 10.15 (21.83) | -1.65 (28.3) | 11.8 (-4.38 - 27.98) |
| LDL-c (mg/dL) | 6.1 (15.66) | -3.6 (24.82) | 9.7 (-3.58 - 22.98) |
| HDL-c (mg/dL) | 1.65 (4.97) | 3.3 (6.63) | 1.65 (-2.1 - 5.4) |
| FBG (mg/dL) | -2.2 (5.75) | 2.45 (6.83) | 4.65 (0.61 - 8.69) |
| HbA1c (%) | -0.12 (0.17) | -0.06 (0.12) | 0.06 (-0.03 - 0.15) |

Data are expressed as mean (SD) change (difference between the final and the baseline values) for all qualitative variables. The last column presents the difference between changes for the control and walnut groups.

BMI – body mass index; CI – confidence interval; DBP – diastolic blood pressure; FBG – fasting blood glucose; HbA1c – glycosylated hemoglobin A1c; HDL-c – high-density lipoprotein cholesterol; LDL-c – low-density lipoprotein cholesterol; SBP – systolic blood pressure; SD – standard deviation; s-VCAM-1 – soluble vascular cell adhesion molecule-1; TC – total cholesterol; TG – triglycerides.

**Table S5.** Linear mixed models predicting change (difference between final and baseline values) in outcome variables, in function of the treatment, adjusted for period, with interactions, and with random effects for patients.

| Dependent variable | Intervention (95% CI) | P | Period (95% CI) | P | Intervention:Period (95% CI) | P |
|---|---|---|---|---|---|---|
| Waist circumference (cm) | -1.22 (-2.44 - -0.01) | 0.049 | 0.14 (-1.07 - 1.36) | 0.814 | 1.08 (-0.85 - 3.02) | 0.264 |
| Hip circumference (cm) | -0.73 (-1.81 - 0.36) | 0.181 | -0.73 (-1.81 - 0.36) | 0.181 | 1.01 (-0.83 - 2.85) | 0.272 |
| Waist-hip ratio | -0.01 (-0.02 - 0.01) | 0.308 | 0.01 (0 - 0.02) | 0.233 | 0 (-0.02 - 0.02) | 0.731 |
| Body weight (kg) | -0.72 (-2.04 - 0.6) | 0.275 | -1.01 (-2.33 - 0.31) | 0.129 | 1.21 (-0.66 - 3.08) | 0.197 |
| BMI (kg/m$^2$) | -0.26 (-0.7 - 0.18) | 0.235 | -0.38 (-0.82 - 0.06) | 0.089 | 0.44 (-0.2 - 1.08) | 0.168 |
| Body fat mass (%) | 0.05 (-1.23 - 1.34) | 0.933 | -0.86 (-2.15 - 0.42) | 0.18 | 0.73 (-1.49 - 2.95) | 0.507 |
| Body water (%) | 0.31 (-0.73 - 1.35) | 0.551 | 0.93 (-0.11 - 1.97) | 0.079 | -0.91 (-2.6 - 0.77) | 0.278 |
| Visceral/abdominal fat rating | -0.15 (-0.6 - 0.3) | 0.513 | -0.29 (-0.74 - 0.16) | 0.196 | 0.16 (-0.57 - 0.89) | 0.656 |
| s-VCAM-1 (ng/mL) | 2.87 (-114.24 - 119.98) | 0.961 | 16.61 (-100.5 - 133.71) | 0.775 | 14.89 (-155.51 - 185.29) | 0.86 |
| SBP (mmHg) | -1.21 (-10.78 - 8.35) | 0.798 | -0.3 (-9.87 - 9.26) | 0.949 | -6.6 (-20.29 - 7.1) | 0.335 |
| DBP (mmHg) | -1.62 (-10.57 - 7.34) | 0.716 | -2.07 (-11.03 - 6.88) | 0.641 | -1.31 (-13.98 - 11.35) | 0.834 |
| TG (mg/dL) | -32.12 (-74.09 - 9.85) | 0.129 | -15.21 (-57.18 - 26.76) | 0.466 | 26.67 (-35.42 - 88.75) | 0.389 |
| TC (mg/dL) | -18.26 (-41.73 - 5.2) | 0.123 | -4.17 (-27.64 - 19.29) | 0.72 | 13.43 (-19.75 - 46.62) | 0.416 |
| LDL-c (mg/dL) | -12.16 (-31.42 - 7.1) | 0.208 | 2.2 (-17.06 - 21.47) | 0.818 | 5.96 (-21.28 - 33.2) | 0.659 |
| HDL-c (mg/dL) | 0.37 (-5 - 5.75) | 0.888 | -3.26 (-8.64 - 2.11) | 0.226 | 2.11 (-5.49 - 9.71) | 0.576 |
| FBG (mg/dL) | 4.24 (-0.68 - 9.17) | 0.089 | 5.7 (0.77 - 10.62) | 0.025 | 2.17 (-5.95 - 10.29) | 0.59 |
| HbA1c (%) | 0.01 (-0.12 - 0.13) | 0.933 | -0.17 (-0.29 - -0.05) | 0.008 | 0.09 (-0.11 - 0.28) | 0.375 |

Data are expressed as mean (SD) for all quantitative variables. BMI – body mass index; CI – confidence interval; DBP – diastolic blood pressure; FBG – fasting blood glucose; HbA1c – glyco-sylated hemoglobin A1c; HDL-c – high-density lipoprotein cholesterol; LDL-c – low-density lipo-protein cholesterol; SBP – systolic blood pressure; SD – standard deviation; s-VCAM-1 – soluble vascular cell adhesion molecule-1; TC – total cholesterol; TG – triglycerides.
